# Smart Cellulose-Based Janus Fabrics with Switchable Liquid Transportation for Personal Moisture and Thermal Management

**DOI:** 10.1007/s40820-024-01510-5

**Published:** 2024-09-26

**Authors:** Jianfeng Xi, Yanling Lou, Liucheng Meng, Chao Deng, Youlu Chu, Zhaoyang Xu, Huining Xiao, Weibing Wu

**Affiliations:** 1 Jiangsu Co-Innovation Center of Efficient Processing and Utilization of Forest Resources, International Innovation Center for Forest Chemicals and Materials, Jiangsu Provincial Key Lab of Sustainable Pulp and Paper Technology and Biomass Materials, Nanjing Forestry University, Nanjing, 210037 People’s Republic of China; 2 College of Materials Science and Engineering, Nanjing Forestry University, Nanjing, 210037 People’s Republic of China; 3 Department of Chemical Engineering, University of New Brunswick, Fredericton, NB E3B 5A3 Canada; 4 Macromolecular Chemistry and Bavarian Polymer Institute, University of Bayreuth, Bayreuth, 95440 Germany

**Keywords:** Directional water transport, Cotton fabric, Anti-gravity directional liquid transportation, Janus wettability

## Abstract

The Janus fabrics designed for personal moisture/thermal regulation have garnered significant attention for their potential to enhance human comfort. However, the development of smart and dynamic fabrics capable of managing personal moisture/thermal comfort in response to changing external environments remains a challenge. Herein, a smart cellulose-based Janus fabric was designed to dynamically manage personal moisture/heat. The cotton fabric was grafted with N-isopropylacrylamide to construct a temperature-stimulated transport channel. Subsequently, hydrophobic ethyl cellulose and hydrophilic cellulose nanofiber were sprayed on the bottom and top sides of the fabric to obtain wettability gradient. The fabric exhibits anti-gravity directional liquid transportation from hydrophobic side to hydrophilic side, and can dynamically and continuously control the transportation time in a wide range of 3–66 s as the temperature increases from 10 to 40 °C. This smart fabric can quickly dissipate heat at high temperatures, while at low temperatures, it can slow down the heat dissipation rate and prevent the human from becoming too cold. In addition, the fabric has UV shielding and photodynamic antibacterial properties through depositing graphitic carbon nitride nanosheets on the hydrophilic side. This smart fabric offers an innovative approach to maximizing personal comfort in environments with significant temperature variations.

**Supplementary Information:**

The online version contains supplementary material available at 10.1007/s40820-024-01510-5.

## Highlights

- A smart all-cellulose Janus fabric was designed for personal moisture/thermal management.
- The fabric can dynamically and continuously control the liquid transportation time in response to the temperature.
- The fabric can accelerate the heat dissipation rate at high temperatures, while slow it down at low temperatures.

**Supplementary Information:**

The online version contains supplementary material available at 10.1007/s40820-024-01510-5.

## Graphical abstract

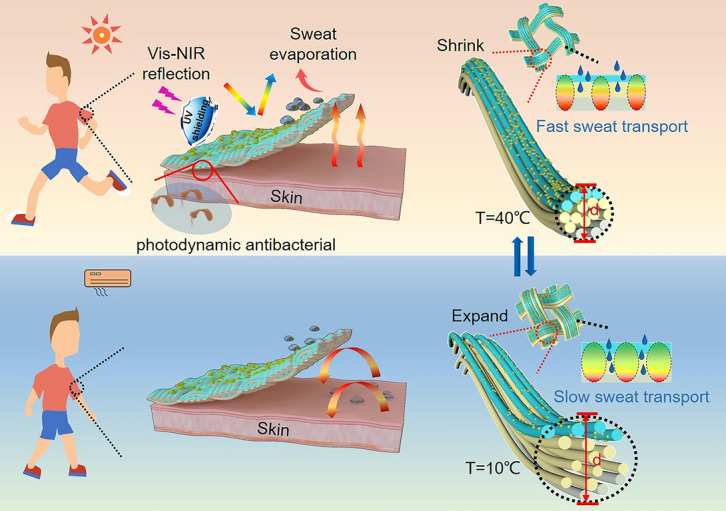

## Introduction

With the improvement of living quality and lifestyle concepts, people have increasingly high demands for the comfort of clothing [1–3]. Clothing worn in high-temperature environments should not only provide a cooling sensation, but also avoid producing a sticky feeling after the body sweats [4–6]. It is well known that evaporation is the main way to dissipate heat for human [7, 8]. The efficient transporting sweat of fabrics, which is able to accelerate evaporation, is the key to improve the cooling effect of clothing [9, 10]. Janus structural materials with the anisotropic wettability have been reported to enable directional water transport [11]. This unique structure efficiently manages body moisture and regulates heat by facilitating sweat transportation and evaporation. Additionally, it prevents stickiness post-sweating, thereby significantly enhancing the overall comfort of wearing the clothing [12–14].

In recent years, various fabrics with Janus structures have been developed for potential applications in moisture wicking [8, 15, 16]. Initially, a lot of research were carried out on the Janus fabrics structural design, such as plasma etching [17], laser perforation [18, 19] and electrospinning [20, 21] to construct the gradient wettability channels. With the advancement of technology, various functional particles were added to further improve the cooling performance of the fabric by utilizing the infrared (IR) radiation and reflecting sunlight of the particles to cooperate with the Janus structure [22–24]. At the same time, the Janus structure is endowed with more diverse properties, such as asymmetry moisture (water vapor) transport ability [8] and sweat sensing monitoring [25]. In addition to the design of the Janus structure and multifunctional applications of the fabric, the development and application of fabrics in specific environments also need to be concerned. For example, when the human body suddenly transitions from a hot outdoor environment to a significantly cooler indoor setting, particularly in scenarios where individuals engage in summer activities and subsequently enter air-conditioned spaces, or individuals required to work in an ice storage room, under such inadvertent temperature drops, the rapid discharge of moisture can make the body feel supercooled and cause discomfort. Although there are some reports that makes Janus fabric with switchable wettability on the side close to the skin, changing from hydrophobic to hydrophilic at low temperatures [10], so as to lock in moisture and maintain human temperature, this can also trap a large amount of sweat and prevent it from being expelled, thus increasing the stickiness of the fabric. Therefore, it is particularly essential to regulate the Janus fabric water transport speed to avoid excessive transport in cold conditions. However, the existing reports have not focused on the control of Janus fabrics liquid transport speed at different temperatures.

The stimulus–response polymers can change their shape and size in response to external stimuli, such as pH, temperature or light, based on protonation and deprotonation, hydrogen bond creation and breaking, and changes in molecular configuration [26]. Through grafting the temperature-sensitive polymer onto the yarn, it is expected to regulate the fabric pore structure by changes in yarn shape and size across various temperatures, ultimately controlling the water transport speed [27]. In this work, we demonstrated a smart cellulose-based Janus fabric that can control the water transport speed based on temperature changes for personal moisture/thermal management. This fabric has a three-layer structure, and after the middle layer is grafted with *N*-isopropylacrylamide (NIPAM), the yarn can shrink and expand at different temperatures depending on the unique winding structure between cotton fibers, thus endowing the cotton fabric (CF) pore structure with temperature-responsive characteristics. Meanwhile, hydrophobic ethyl cellulose (EC) and hydrophilic cellulose nanofiber (CNF) are utilized to achieve gradient wettability of yarns. EC and CNF can be well bonded to the yarn surface without falling off through strong hydrogen bonding [28, 29]. Graphitic carbon nitride (g-C_3_N_4_) was added to the hydrophilic side to endow the fabric with UV shielding and photodynamic antibacterial properties. Through smartly controlling the sweat evaporation rate by the water transport speed regulating, rapid heat dissipation at high temperatures and thermal preservation at low temperatures of the fabric can be achieved. This effectively prevents the individuals from feeling supercooled during sudden temperature drops after sweating. The smart cellulose-based fabric has the potential to dynamically regulate human moisture and heat, making it highly suitable for use in the environment with large temperature differences.

## Material and Methods

### Materials

Hydrophilic CF is purchased at local markets. Before use, it was soaked in 1% NaOH for 1 h and cleaned with water and ethanol. CNF was purchased from Guilin Qihong Technology Co., Ltd., H_2_O_2_, ascorbate (H_2_A), EC and urea were obtained from Nanjing Chemical Reagent Co., LTD. Citric acid (CA), sodium hypophosphite (SHP) and N-isopropyl acrylamide (NIPAM) were purchased from Sigma-Aldrich. NaCl, NH_4_OH, NaOH, acetic acid and lactic acid were purchased from Nanjing Chemical Reagent Co., LTD. NIPAM was purified after recrystallization and filtration, the rest of the reagents are used directly without purification.

LB Nutrient agar was purchased from Qingdao Hope Bio-Technology Co., Ltd. Composition: tryptone, 10.0 g; yeast extract powder, 5.0 g; NaCl, 10.0 g; agar, 15.0 g; pH, 7.0 ± 0.2. Before use, 40 g LB Nutrient agar was dissolved in 1 L water.

LB Broth was purchased from Qingdao Hope Bio-Technology Co., Ltd. Composition: tryptone, 10.0 g; yeast extract powder, 5.0 g; NaCl, 10.0 g; pH, 7.0 ± 0.1. Before use, 25 g LB Broth was dissolved in 1 L water.

### Preparation of Thermal Responsive Fabrics

5 g CF was immersed in 10% NIPAM solution at 200 rpm with a liquid ratio of 1:30 under nitrogen atmosphere. After 20 min, 0.075 mL 30% H_2_O_2_ was added and reacted for 2 h. Then, 0.22 g H_2_A was added and reacted for 1 h. The CF after the reaction is thoroughly washed with water and ethanol and then air-dried. The CF modified by PNIPAM is named NCF. The grafting ratio G was calculated according to the following equation:

1 $$\text{G}=\frac{{m}_{1}-{m}_{0}}{{m}_{0}}$$

where ${m}_{0}$ is the weight of CF, and ${m}_{1}$ is the weight of NCF.

### Preparation of Smart Cellulose-Based *Janus* Fabrics

10% EC ethanol solution was sprayed on the one side of the NCF, and the spray jet angle and a beam width were fixed at 90° and 3 cm, respectively, while the spray distance was 5 cm. Before spraying, EC ethanol solution with a total mass 1.5 times the mass of the cotton fabric was weighed. The spraying process is divided into five stages, with each stage applying one-fifth of the total EC solution required. After each spraying, the fabric was dried at 60 °C to remove ethanol immediately. 0.025 g CNF and 0.0125 g g-C_3_N_4_ nanosheets were dispersed in 50-mL water. Then, 0.004 g CA and 0.001 g SHP were added to prepare the hydrophilic coating. The hydrophilic coating was sprayed on the other side of the NCF in the same way as described above. When the weight of the NCF has increased to four times the original, the spraying operation is completed. The fabric was dried at 60 °C. Subsequently, the fabric was kept at 150 °C for 10 min. The final prepared fabrics are named NCFB (fabrics without hydrophilic side), NCFH (fabrics without hydrophobic side), NCFBM (fabrics sprayed with excessive EC) and JCF, and the composition of the final prepared fabric is shown in Table S1.

### Photocatalytic Antibacterial Performance

Bacteria: E.* Coli* and S.* aureus* were obtained from China Center of Industrial Culture Collection.

Bacteria culture: LB Broth solution was used to prepare 3% bacterial suspension. Then, the suspension was placed at 37 °C for 12 h at 200 r min^−1^.

Determination of photocatalytic antibacterial performance: Before test, 5 g of cut fabric was sterilized in an autoclave at 121 °C for 20 min. 15 mL LB Nutrient agar solution at 45 °C was poured into the culture medium, shook well clockwise and left to cool. The normal saline was used as culture medium and the bacterial suspension was diluted to 1.0 × 10^5^ (CFU) mL^−1^. The cut fabric was put into 50 mL of diluted bacterial solution and irradiated under 300 W (*λ* > 420 nm) xenon lamp light source for 60 min. Then, 100 μL irradiated bacterial solution was evenly spread on culture medium. The medium was sealed and placed in an incubator at 37 °C for 24 h.

To calculate growth inhibition rate (GIR), the irradiated bacterial solution was diluted to ensure that the number of colonies is between 30 and 300. Then, the number of colonies were recorded and the growth inhibition rate (GIR) was calculated according to the following equation:

2 $$ {\text{GIR}} = \left( {1 - \frac{{\text{A}}}{{\text{B}}}} \right) \times 100\% $$

where $A$ is the number of colonies cultured from JCF, $B$ is the number of colonies cultured from CF.

### Characterization

The morphology of the fabric was observed with a field emission scanning electron microscope (SEM, JSM-7600F, Hitachi, Japan) at an accelerating voltage of 15 kV. Before SEM imaging, samples were sputter-coated with gold for 90 s. The SEM images were analyzed by ImageJ software to quantitatively evaluate the change of pore size. The morphology of g-C_3_N_4_ nanosheets and CNF was obtained by transmission electron microscope (JEM-1400, Nippon Electronics Co, Japan). TEM images of g-C_3_N_4_ nanosheets and CNF are showed in the supporting material (Fig. S1). Attenuated total reflectance Fourier-transform infrared spectroscopy (ATR-FTIR) was detected on an infrared spectrometer (Vertex 80 V, Brukeroptics, Germany). X-ray photoelectron spectroscopy (XPS) was applied to investigate the surface chemical compositions, performed on an ESCALAB 250 system (Thermo, USA) using an Al Ka X-ray source (1486.6 eV) operated at 50 eV. Air permeability of the fabrics was determined according to GB/T 5453 via a Fully Automate Permeability Instrument (YG461E-III, Ningbo Textile Instrument Factory, China), the test area is 20 cm^2^, and pressure differential is 100 Pa.

Infrared thermal images were taken with an infrared thermal imager (FOTRIC 325pro, Fotric, China). The fabrics were placed on the arm. The arm was placed in a controlled temperature chamber (DNP-9052BS-III, CIMO, China) and photographed after 5 min. To test infrared thermal imaging under sunlight, the arm with the fabric was put under the xenon lamp (CEL-HXUV300, Beijing China Education AuLight Technology (CEAuLight) Co., Ltd., China) at distance of 30 cm with the optical power density of 100 mW cm^−2^.

Water contact angle (WCA) of the fabric was measured by Contact Angle Meter (T200-Auto3 Plus, Biolin Scientific, Sweden). The fabrics were cut into strips and hung in the air by fixing two ends. The water droplet was placed on the fabric surface to test its WCA. When testing the wettability of NCF, the data of water droplets at 3 s on the surface of NCF are selected. When measuring the directional water transport performance, in order to maintain temperature stability, the fabric was placed in a 40 °C oven, an indoor environment, and a 10 °C cool chamber for 5 min, respectively, and then the measurements were conducted directly with necessary insulation measures. The artificial sweat was prepared according to International Organization for Standardization (ISO) 3160–2, containing NaCl (20 g liter^−1^), NH_4_OH (17.5 g liter^−1^), acetic acid (5 g liter^−1^), and lactic acid (15 g liter^−1^), with the pH adjusted to 4.7 by NaOH. It was used to test the directional transport performance.

The water evaporation rate was measured according to GB/T 21655.1. The water evaporation rate ${\text{E}}_{i}$ is calculated as follows:

3 $${\text{E}}_{i}=\frac{m-{m}_{i}}{{m}_{i}-{m}_{0}}\times 100$$

where $m$ is the weight of the fabric moistened by dripping water, and ${m}_{i}$ is the weight of the fabric at a certain time after wetting by dripping water, ${m}_{0}$ is the weight of the original fabric.

The wicking height was measured based on the AATCC TM 197. The sample was cut into 2 cm wide strips, dipped in deionized water with blue dye, and the capillary rise height was recorded.

The optical reflectivity in the sunlight wavelength range of the fabric was measured using a UV–vis-NIR (0.3–2.5 μm) spectrophotometer (UV 3600, Shimadzu, Japan). The surface solar reflectivity $\text{R}$ is calculated as follows:

4 $$ {\text{R}} = \frac{{\mathop \smallint \nolimits_{0.3}^{2.5} R\left( \lambda \right)I\left( \lambda \right)d\lambda }}{{\mathop \smallint \nolimits_{0.3}^{2.5} I\left( \lambda \right)d\lambda }} $$

where $\lambda$ is the wavelength, $R\left(\lambda \right)$ is the surface’s spectral reflectance at wavelength $\lambda$, and $I\left(\lambda \right)$ is AM 1.5 global solar spectral irradiance.

The UV–Vis transmittance of each fabric was measured using a UV4100 spectrophotometer (Hitachi Ltd., Tokyo, Japan) attached to a 116JO321 integrating sphere (Hitachi Ltd., Tokyo., Japan). The UVA, UVB blocking percentages were calculated as follows:

5 $$  {\text{UVA}}\;{\text{blocking}} = 100\%  - \frac{1}{m}\sum\limits_{{315}}^{{400}} {T_{i} } \left( \lambda  \right)  $$

6 $$  {\text{UVB}}\;{\text{blocking}} = 100\%  - \frac{1}{k}\sum\limits_{{290}}^{{315}} {T_{i} } \left( \lambda  \right) $$

where ${T}_{i}(\lambda )$ is the spectral transmittance of sample at wavelength $\lambda$, $m$ and $k$ are the number of measurements between 315–400 nm and 290–315 nm, respectively.

## Results and Discussion

### Fabrication and Structure of Smart Cellulose-Based Fabrics

**Fig. 1:**
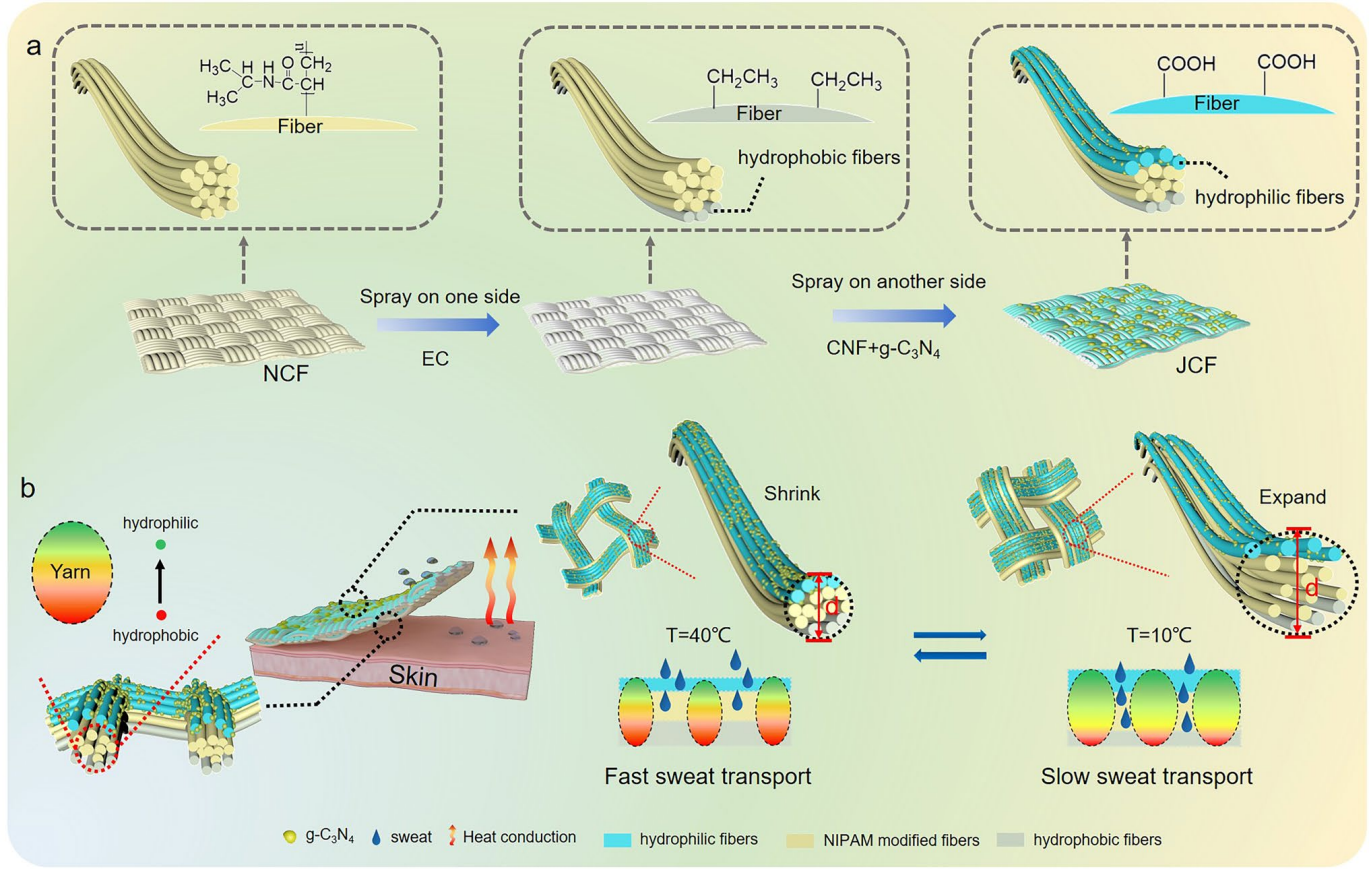
**a** Fabrication of JCF **b** Schematic diagram of the smart fabric with Janus wettability for personal moisture and thermal management

It is necessary to develop a smart fabric that can be able to wick quickly in hot weather to lower body temperature and slowly to avoid supercooling when suddenly entering a cold environment after sweating. In this work, we designed a three-layer smart fabric with Janus wettability and temperature-responsive controllable pore size (Figs. 1a and S2). Through PNIPAM modification, the fabric middle layer can realize pore size dynamical regulation. In addition, wettability gradients were constructed by using different hydrophilic/hydrophobic cellulose materials on both sides of the fabric. The bottom (close to the skin) of the fabric was sprayed with EC to create the hydrophobic surface, and the top of the fabric was sprayed with CNF and g-C_3_N_4_ nanosheets. Because of the carboxyl group present on the surface of CNF, it exhibits better hydrophilicity compared to cotton fabric. The deposition of g-C_3_N_4_ nanosheets endows the fabric with the UV shielding ability, high solar reflectivity, and photodynamic antibacterial properties. Subsequently, the modification of cellulose by citric acid further enhances the hydrophilicity of the fabric top and forms crosslinked network that makes CNF and g-C_3_N_4_ nanosheets firmly loaded on the yarn [30]. This method of spraying different hydrophilic/hydrophobic cellulose materials imparts a wettability gradient to the yarn in the fabric, enabling the discharge of sweat (Fig. 1b). Besides, the JCF has different liquid transport speeds at different temperatures due to the presence of thermo-sensitive fabric middle layer. In the cold environment, the yarn in the JCF will expand at low temperature because of the grafted PNIPAM, and the water vapor evaporation slows down, the JCF can lock the heat and play a heat preservation role. On the contrary, the fabric pore size increases and its moisture-wicking performance is improved as the temperature rises. This smart cellulose-based Janus fabric with controlled sweat transport speed, UV shielding and antibacterial properties can be applied to human moisture management during sudden temperature changes.

**Fig. 2:**
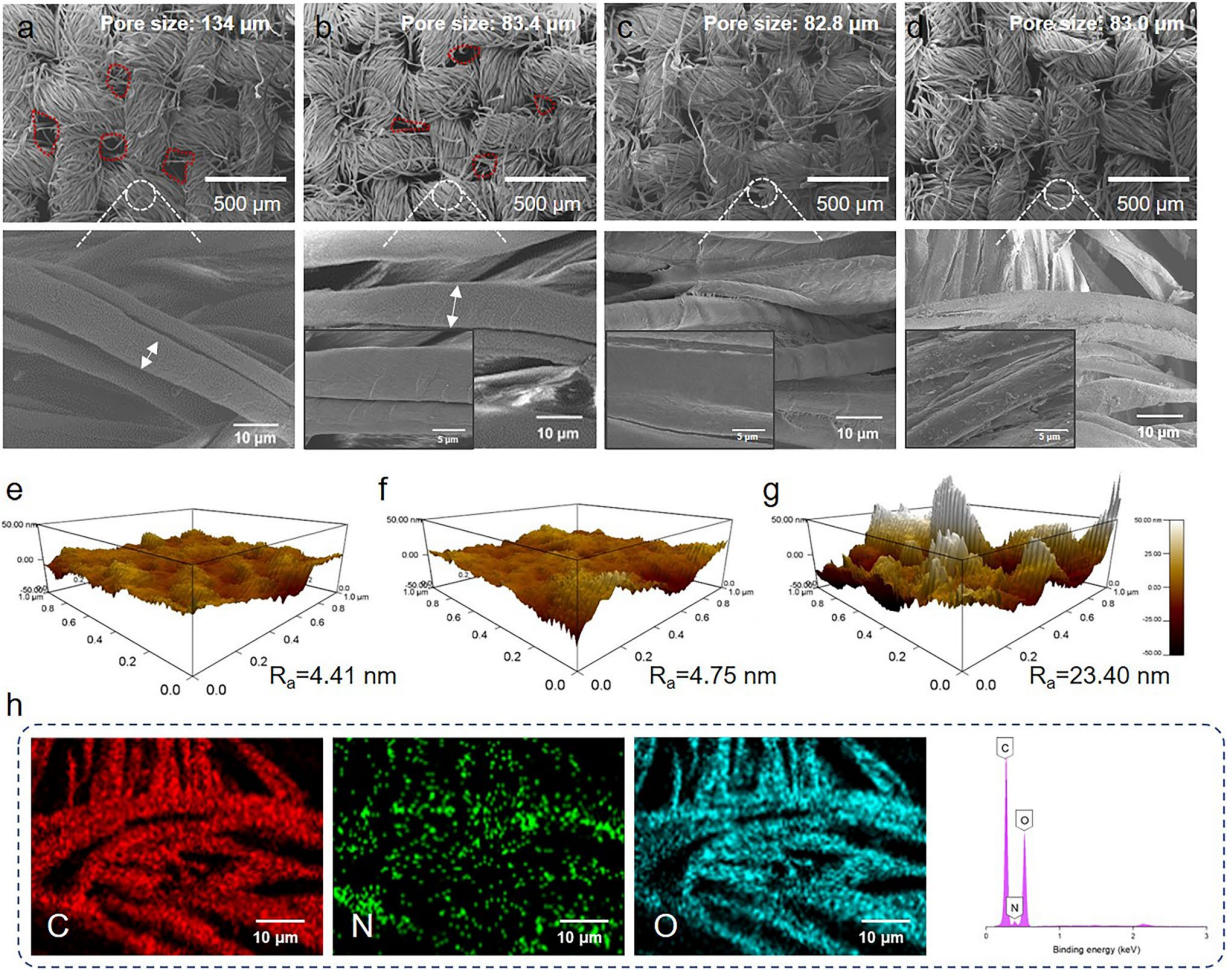
SEM image of **a** CF, **b** NCF, **c** hydrophobic side of JCF, and **d** hydrophilic side of JCF. The surface roughness of the fiber on **e** NCF, **f** JCF-without C N and **g** JCF hydrophilic side. **h** Element mapping image and EDS spectra of JCF hydrophilic side

The pore size of fabric is very important for sweat transport. The average pore size of CF is 134 ± 2.5 μm (Fig. 2a). After the PNIPAM grafting, there is a slight increase in the size of individual cotton fibers (Fig. 2b), reducing the pore size of NCF to 83.4 ± 1.2 μm. FTIR and XPS also confirm the presence of grafted PNIPAM (Fig. S3, Table S2). Additionally, as EC and CNF are sprayed on the surface of the yarn, the overall pore structure of the fabric generally remains unchanged (Fig. 2c, d). On the hydrophobic side, it can be seen that the gaps between fibers are filled with EC. At the same time, there is an increase in the roughness of the fiber surface on the hydrophilic side (Fig. 2e–g). The surface roughness *R*_a_ value of cotton fiber on NCF is 4.41 nm, and the *R*_a_ value increases to 4.75 nm after spraying CNF. Due to the stacking of g-C_3_N_4_ nanosheets, the roughness of the cotton fiber is further improved to 23.40 nm. This roughness improvement is beneficial to improve the hydrophilicity of the fiber [31] (Fig. S4). In addition, EDS element distribution analysis reveals that the N element is evenly distributed on the surface of the hydrophilic side, indicating the uniform deposition of g-C_3_N_4_ nanosheets on the CNF surface (Fig. 2h). The surface of the NCF after PNIPAM grafting has a WCA of 40.2° at 20 °C, and when the temperature increases to 40 °C, its WCA increases to 72.5° (Fig. S5). Reason can be ascribed to the increasing association degree between the hydrophobic isopropyl groups on the PNIPAM chain at high temperature [32].

Human joints and intense exercise will cause the fabric deformation, which may further lead to the structural damage. For JCF, the sprayed EC, CNF, and g-C_3_N_4_ nanosheets are well fixed on the yarn surface without falling off under being twisted, stretched, rubbed and stabbed. At the same time, the fabric still has directional water transport performance (Fig. S6). In the tri-s-triazine structure of g-C_3_N_4_ nanosheets, each nitrogen atom possesses a lone pair of electrons, which will form hydrogen bonds with the -OH and -COOH groups present on the CNF [33]. Besides, the covalent network under the citric acid crosslinking further fixed CNF and g-C_3_N_4_ nanosheets firmly [34]. Under the action of ultrasonic and stirring, g-C_3_N_4_ nanosheets demonstrate strong adhesion to the fibers, while EC remains well attached to the interstitial spaces between fibers due to its hydrophobic protection, suggesting the washable property of the fabric.

### Temperature-Controlled Directional Water Transport

**Fig. 3:**
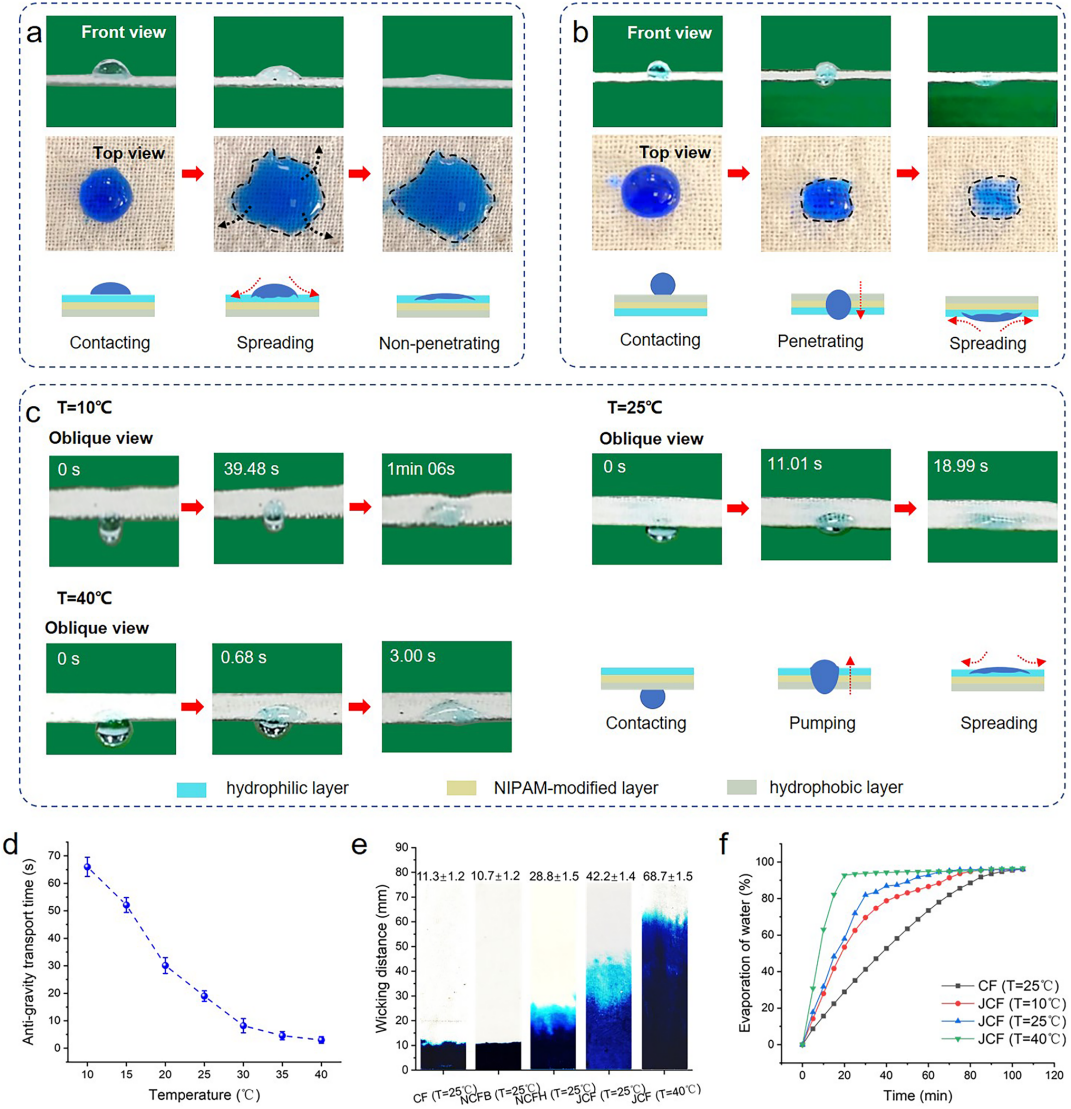
**a** Water dripped onto the JCF hydrophilic side at 25 °C. **b** Water dripped onto the JCF hydrophobic side at 25 °C. **c** Water droplet vertically transporting from hydrophobic side (contact with the skin) to the hydrophilic side (exposure to ambient) at 10 °C, 25 °C and 40 °C. **d** Antigravity water transport time of JCF at different temperatures. **e** The wicking distance of the fabrics. **f** Water evaporation of CF and JCF at 10 °C, 25 °C and 40 °C

The wettability gradient generated by the hydrophilic/hydrophobic difference on both sides of the fabric drives the liquid to move irreversibly from the hydrophobic side to the hydrophilic side [35–37]. Water droplets were placed on both sides of the JCF to observe its directional water transport performance. When the water droplet touches the hydrophilic side, it cannot penetrate to the other side (Fig. 3a). However, when it touches the hydrophobic side, the water droplet can penetrate vertically to the other side (Fig. 3b). From the top view, on the JCF hydrophilic side, the spherical water droplets rapidly spread out horizontally without longitudinal penetration.

The wettability behaviors are further detected on both sides of the JCF. For the JCF hydrophilic side, the water droplet spread out on the surface, and the red direction line shifts significantly outward during the permeation process (Fig. S7a). However, on the hydrophobic side, the red directional line goes straight down and the droplets penetrate vertically into the fabric (Fig. S7b). These results suggest that the wettability of the two sides of the JCF is opposite, thus providing a driving force for the directional transport of moisture.

The fabric exhibits excellent anti-gravity directional water transport, which is expected to enable the transfer of sweat from the side of the fabric near the skin to the side of the fabric exposed to air. It is worth noting that this anti-gravity directional water transport can exhibit different transport speeds at different temperatures. The water droplet that contact the hydrophobic side vertically from the bottom take 1 min 06 s to fully pump to the hydrophilic side at 10 °C, 18.99 s at 25 °C, and only 3.00 s at 40 °C (Fig. 3c). With the increase in temperature, the time taken for anti-gravity transmission decreases gradually (Fig. 3d). This shows that fabrics can achieve smart, continuous and dynamic management of sweat transport speed at different temperatures. At the same time, the decrease in water transport time becomes less noticeable when the temperature is higher than 30 °C. Crosslinked PNIPAM has a lowest critical solution temperature (LCST) at 32 °C. When the temperature is higher than 32 °C, most of the PNIPAM chains shrink, causing the transport time to plateau even as the temperature rises further [38]. Besides, the transition for JCF is not as “abrupt” as that reported in some research since the comparatively low grafting degree (The grafting ratio is 15.1 ± 0.2%) [39].

Rapid water transport at high temperatures is important. The existing reports have evaluated the water transport of Janus fabrics, but has not quantified it [8, 40]. In this work, the water transport performance of the fabric is quantitatively evaluated by the time of the water droplet transport from hydrophobic side to hydrophilic side. Most of the existing reports have measured the water transport performance at room temperature, and the prepared fabrics cannot realize the control of water transport speed at different temperatures. The JCF prepared in this work can realize the dynamic control of water transport speed in the temperature range of 10–40 °C. The water transport speed at high temperature (30–40 °C) is basically higher than those reported in other works [8, 17, 40, 41], as shown in Fig. S8. At the same time, JCF can slow down the transport speed when the temperature drops sharply to lower than 20 °C. Briefly, the enhanced transport speed is able to facilitate the rapid sweat discharge on the skin surface in hot weather, and the decrease in transport speed at the low temperature can prevent the discomfort of the human body when it suddenly enters the cold environment.

Good hygroscopic fabrics are able to readily collect perspiration from people and release heat through evaporation, assisting in the regulation of body temperature. The hygroscopicity of fabrics was compared by the wicking distance. Compared to CF and NCFB, the wicking distance of NCFH increase to 28.8 ± 1.5 mm (Fig. 3e). This indicates that the presence of hydrophilic side can improve the moisture absorption of fabric and promote the sweat absorption of fabric. The wicking distance is further increased to 42.2 ± 1.4 mm. The pumping effect of the JCF on water in thickness can promote the water transfer to the fabric hydrophilic side, and at the same time, the water will spread out rapidly on the hydrophilic side. When the temperature rises from 25 to 40 °C, the wicking distance of JCF is further increased to 68.7 ± 1.5 mm due to the increase in the fabric pore size.

In practical application, the dynamic adjustment of fabric to water evaporation rate can achieve thermal management. The fabric quick-drying performance was evaluated by the water evaporation rate. The water evaporation rate of JCF at 25 °C is 45 min shorter than that of CF (Fig. 3f). Besides, the temperature responsiveness of JCF can adjust the water evaporation rate based on temperature. The water evaporation rate of JCF at 40 °C is 3.25 times higher than that at 25 °C, whereas it takes 1.3 times longer at 10 °C than that at 25 °C. This fascinating water evaporation performance of JCF is attributed to its excellent directional water transport property and rapid moisture absorption ability. Due to the good hygroscopic property and its wetting gradient channel, the liquid can be pumped from the inside of the fabric to the hydrophilic side of the fabric [42, 43]. In addition, the liquid can spread on the surface quickly rely on the strong wettability of the hydrophilic side, resulting in a lower moisture mass per unit area of the fabric [44], thereby promoting faster evaporation of water. Besides, the high water transport speed at high temperature is able to prevent water from staying too long inside the fabric, and further promoting the spreading and evaporation of water on the hydrophilic side. The JCF air permeability is still ≥ 150 mm s^−1^ even after water wetting (Fig. S9). This shows that the fabric still has good breathability even when wet with sweat.

### Mechanism of Smart Directional Water Transport

**Fig. 4:**
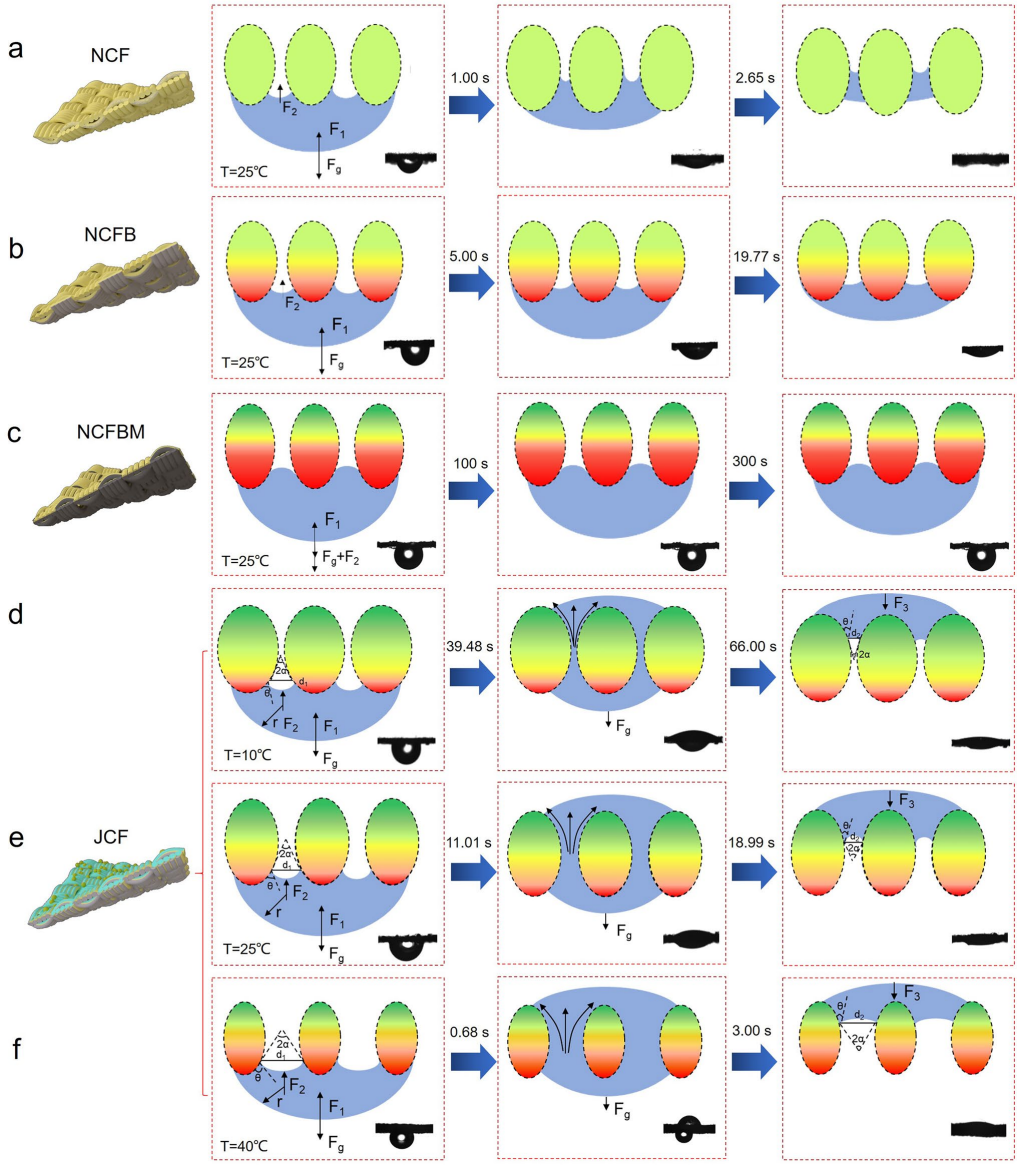
**a-c** Antigravity directional water transport performance and diagram of directional water transport mechanism of different fabrics at 25 °C. Diagram of antigravity directional water transport mechanism at **d** 10 °C, **e** 25 °C and f 40 °C

The structure of the fabric dictates its gradient wettability performance. We tested the anti-gravity directional water transport performance of three fabrics: NCF, NCFB, and NCFBM, and analyzed the role of the fabric structure from a mechanical perspective. The fabric pore structure is formed by the interlacing of the yarns, and the sweat droplets are pumped up between the yarns as they pass through the fabric. When the sweat contacts the fabric, in addition to its own gravity (${F}_{g}$), the droplet is subjected to the water surface tension (${F}_{1}$) and the capillary force (${F}_{2}$) [18]. For NCF, the sweat droplet spreads rapidly and cannot be pumped to the fabric other side (Fig. 4a). In the absence of additional driving force, the sweat droplet remains trapped within the NCF and cannot be discharged. Although the hydrophilic fabric can absorb sweat, it cannot drain sweat to the outside of the fabric. When only EC is sprayed on the NCF one side (fabrics without hydrophilic side), the sweat droplet is spreading lengthwise and are not being pumped to the other side, some water is left at the bottom (Fig. 4b). Due to the lack of wettability gradient, sweat cannot be transferred to the opposite side of the fabric. In addition, the thickness of the hydrophobic side also affects the directional sweat transport (Fig. 4c). Excessive EC spraying results in the increased thickness of the hydrophobic side, which prevents droplet contact with the hydrophilic part of the yarn. At the same time, the droplets are subjected to downward capillary forces and cannot be pumped longitudinally into the interior of the fabric. Therefore, the hydrophobic side should be thin, and the wettability gradient are the key to realize anti-gravity directional water transport of the fabric.

The fabric anti-gravity directional sweat transport speeds at different temperatures depends on the change of fabric pore size. When the surrounding temperature rises from 10 to 40 °C, due to the surface of JCF contains bound water and adsorbed water, a single cotton fiber will become dehydrated and shrink, resulting in a decrease in yarn size and an increase in the pore size (Fig. S10, Movies S1, S2). Figure 4d–f presents the smart anti-gravity directional water transportation mechanism (Movies S3-S5). The water transport in the fabric channel is divided into three stages: 1) initial wetting stage, 2) rapid transmission stage and 3) diffusion stage.

In the initial wetting stage, when the water touches the fabric, the resultant force pushing the droplet upward is as follows [45]:

7 $$ F = F_{1} + F_{2} - F_{g} $$

8 $$ {\text{F}}_{1} \sim {\uppi }\gamma {\text{D}}^{2} /2{\text{r}} $$

9 $$ F_{g} = K \cdot \frac{4}{3}\pi r^{3} \rho g $$

10 $$ F_{2} = \gamma \pi d_{1} \cdot \cos \left| {\theta - \alpha } \right| $$

where $\gamma$ is the surface tension of the water, $\text{D}$ is the diameter of the micropore, $r$ is the radius of the drop, $K$ is the of the drop, $\rho$ is the mass density of the liquid, $g$ is the acceleration of gravity, ${d}_{1}$ is the diameter of the three-phase contact line, $\theta$ is the liquid contact angle, and $2\alpha$ is the cone degree of the capillary channel. In Eq. 1, ${F}_{1}$ and ${F}_{g}$ are equal at both temperatures, but ${F}_{2}$ is different. Because of the PNIPAM modification, ${d}_{1}$ increases at high temperatures. When the temperature increases, $\theta$ is increased, at the same time, $\alpha$ is related to the pore size and increased by changes in the pore size. In addition, as the droplet rises at the same temperature, α is changing accordingly. Although it is difficult to accurately determine the values of $\mid \theta -\alpha \mid$ during liquid transportation process, the faster transportation rate suggests that ${d}_{1}$ may play a more important role, thereby increasing ${F}_{2}$. In the rapid transport stage, water is affected by the wettability gradient and is absorbed continuously into the channel without backflow. In the diffusion phase, water reaches the other side of the fabric and is subjected to downward resistance (${F}_{3}$):

11 $$ F_{3} = \gamma \pi d_{2} \cdot \cos \left| {\theta - \alpha } \right| $$

where ${d}_{2}$ is the diameter of the three-phase contact line and $2\alpha$ is the cone degree of the capillary channel. Similar to the initial wetting stage, the increase in ${d}_{2}$ may lead to greater resistance (${F}_{3}$) at 40 °C. As a result, water droplets are more likely to spread out on the hydrophilic side.

### Moisture/Thermal Management of JCF

**Fig. 5:**
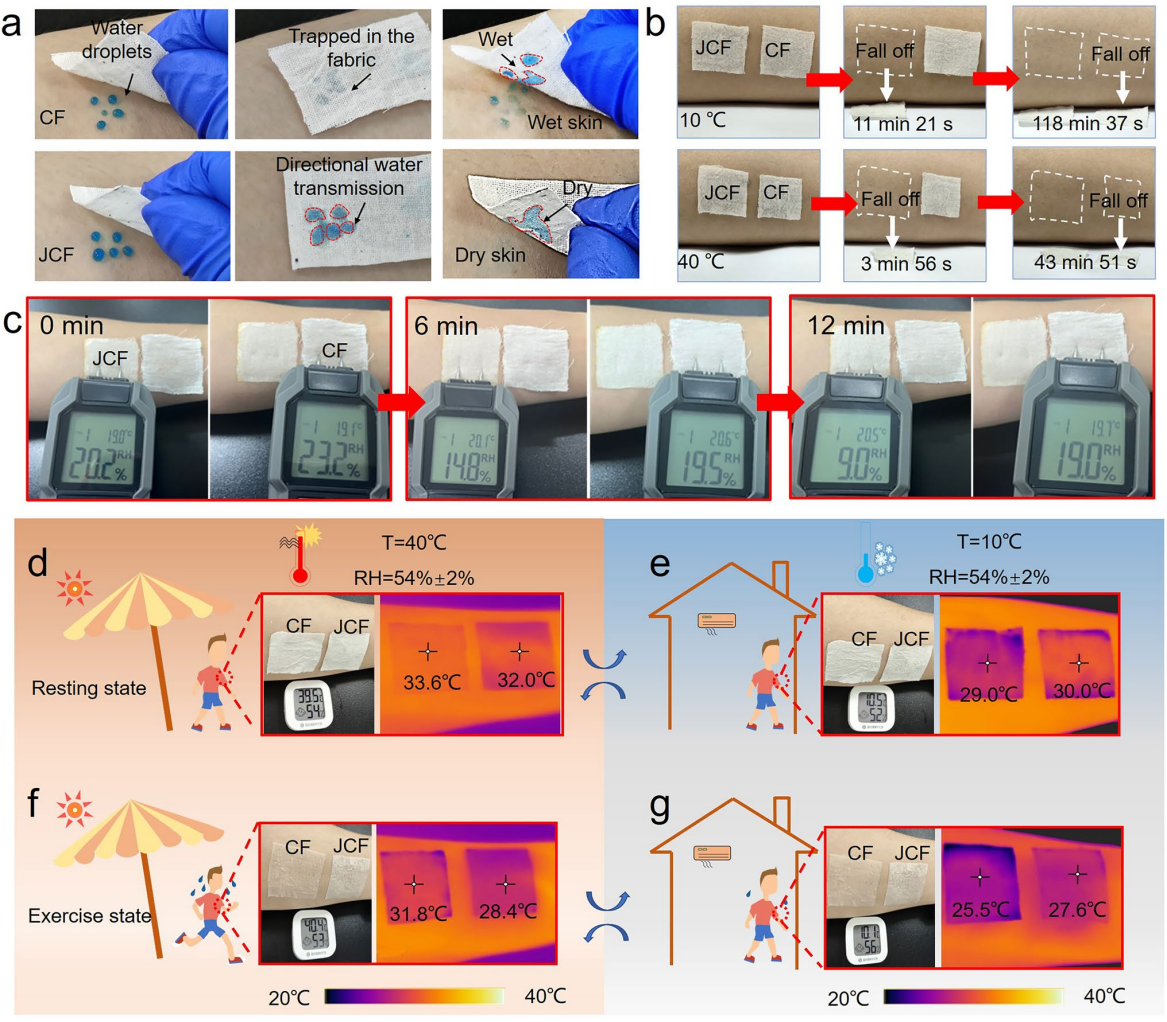
**a** Practical test of the fast moisture-wicking property of the CF and JCF at 20 °C. **b** Determination of the stickiness of fabrics after wetting. **c** Moisture-wicking performance and fast water transportation of cotton (right) and JCF (left) when applied on wet skin at 20 °C. Infrared images taken when fabrics were applied to skin during human resting state at **d** 40°C and **e** 10 °C. Infrared images taken when fabrics were applied to wet skin during human exercise state at **f** 40 °C and **g** 10 °C

The sweat absorbed by clothing may cause it to stick to the skin, thus limiting the movement of the body and producing a feeling of discomfort. This smart cellulose-based Janus fabric with anti-gravity directional liquid transport is very suitable for developing sweat-wicking fabrics. As shown in Fig. 5a, when JCF is applied to human skin surface, blue water droplets can be effectively pumped to the hydrophilic side, which speeds up the drying process for perspiring skin. However, for CF, moisture is locked inside the fabric and the skin surface cannot stay dry, which is not conducive to the evaporation of sweat. To show the characteristics of fast drying and non-sticking of the fabric after sweating more intuitively, JCF and CF surface are sprayed with the same amount of water (20% water content relative to fabric weight) and attached to the human skin. At low temperatures, JCF falls off the skin after 11 min 21 s, while CF takes 118 min 37 s. At high temperatures, JCF only takes 3 min 56 s to fall off the skin (Fig. 5b). Unlike other reports that prevents supercooling by locking moisture inside the fabric [10], JCF can control the evaporation rate of water to prevent clothes from becoming sticky and causing discomfort to the personal body. At the same time, a comparative analysis of moisture content between JCF and CF offers a more intuitive representation of JCF superior moisture-wicking performance. 50 μL artificial sweat was applied to human skin at an ambient temperature of 25 °C and covered with JCF (left) and CF (right), respectively. The surface humidity was measured by a handheld hygrometer. Compared with CF, JCF can reduce the surface moisture from 20.2% to 9.0% within 12 min (Fig. 5c), demonstrating better moisture-wicking performance.

Thermal management at different temperatures is very important for human comfort. The directional liquid transport performance of the fabric also gives the fabric thermal management ability. For high-temperature weather, the anti-gravity directional liquid transport can quickly take away sweat on the skin, and make sweat spread out on the fabric surface and evaporate, ultimately cooling the skin temperature. JCF can accelerate the heat removal in a hot indoor environment (40 °C), and the temperature of JCF is 1.6 °C lower than that of CF (Fig. 5d). There is an increase in the pore size of JCF under high temperatures, which helps dissipate heat. Additionally, the invisible sweating that occurs during human rest can intensify under high temperatures [46]. The Janus structure of JCF can maintain skin dryness and prevent trace amounts of sweat from being kept within the fabric, facilitating the expulsion of damp and hot air. The rapid transport and evaporation of trace amounts of sweat by JCF can effectively and quickly dissipate heat, making it more conducive to heat dissipation in the human body at rest compared to CF. Moreover, when the temperature drops suddenly, JCF exhibits better thermal preservation performance because of the pore size decline, and its temperature can be increased by 1.0 °C compared with CF (Fig. 5e). Because of the grafted PNIPAM on cotton fiber, the pore size of JCF at 10 °C is much smaller than CF, which enables JCF to maintain human body temperature at low temperatures. In addition, when sweat is present on the skin (50 μL artificial sweat was applied to human skin), JCF shows better heat dissipation performance at 40 °C, and the temperature of JCF is 3.4 °C lower than that of CF (Fig. 5f). The sweat on the JCF hydrophilic side surface will spread rapidly, reducing the sweat amount per unit area of the fabric. In this case, the sweat will accelerate evaporation, which will further lower the body temperature. Similarly, when the human body enters the 10 °C environments after plenty of sweating, the insulation effect of JCF is 2.1 °C higher than that of CF (Fig. 5g). When the ambient temperature is lower than the body temperature, radiation, conduction, and convection rather than sweat evaporation mainly contribute to the heat dissipation. JCF can transport sweat from skin to the other side of the fabric. The dryness on the skin side helps to retain body heat. Meanwhile, the reduction in fabric pore size at low temperatures also slows down the loss of heat. When the fabric contains water, the presence of water promotes the PNIPAM chains to more easily present an extended state, making the pore size change more pronounced. In addition, the JCF exhibits superior cooling performance when exposed to sunlight, the temperature is 4.3 °C lower than CF under strong light (Fig. S11a). The CF has a reflectance of only 53.2% in the visible-light region (0.3–2.5 μm), while JCF shows a stronger reflectance of 92.8% (Fig. S11b). This cooling performance is due to the addition of high refractive index g-C_3_N_4_ nanosheets, which improves the light reflection. This smart fabric has better performance in outdoor high-temperature sunlight environment.

### UV Shielding and Photodynamic Antibacterial Performance

The strong ultraviolet light in summer will cause damage to human skin. With the addition of g-C_3_N_4_ nanosheets, the UV shielding properties of the fabric are significantly improved. The UVA and UVB blocking rates of CF were 55.0% and 64.3%, respectively, and the blocking rate of UVA and UVB increased to 75.0% and 75.9%, respectively (Fig. S12). In order to display the UV shielding performance of the fabric more intuitively, the fabrics were covered on the UV-induced color change card and irradiated with 365 nm ultraviolet light at the distance of 15 cm for 5 min. As shown in Fig. S13, JCF can prevent the transmission of ultraviolet light [47], and shows good UV shielding performance.

At the same time, g-C_3_N_4_ nanosheets are able to produce strong oxidation-reducing substances under light, such as ·OH, H^+^, ·O_2_^−^, which show strong bactericidal effect [48, 49]. After 60 min illumination, the growth inhibition rate of S.* aureus* and E.* coli* by JCF reaches 97.17% and 95.12%, respectively (Fig. S14). Different from traditional clothing, JCF integrates excellent anti-gravity directional liquid transport, high solar reflectivity, efficient UV shielding, and photodynamic antibacterial performances in one design, which synergistically contribute to personal wearing comfort.

## Conclusions

In this work, a cellulose-based three-layer fabric with Janus wettability and smartly tunable directional water transport speed has been designed. The CF modified by NIPAM has temperature-responsive pore structure channels. At the same time, hydrophobic EC and hydrophilic CNF are used on both sides of the fabric to impart a wettability gradient, creating a Janus structure. This Janus structure is robust due to strong hydrogen bonding between cellulose. In hot environments, fabrics cool the skin by increasing the pore size and accelerating the evaporation of sweat. When entering a cold environment, the fabric pore size becomes smaller, the evaporation rate of sweat is slowed down, and the human body heat can be maintained while maintaining non-sticky and prevent feeling supercooled. Additionally, g-C_3_N_4_ nanosheets were added to the JCF hydrophilic side to improve the UV shielding properties and impart photodynamic antibacterial performance. This smart fabric has great potential for personal moisture/heat management.

## Supplementary Information

Below is the link to the electronic supplementary material.

Supplementary file1 (DOCX 4329 KB)

Supplementary file2 (MP4 4294 kb)

Supplementary file3 (MP4 4370 kb)

Supplementary file4 (MP4 5437 kb)

Supplementary file5 (MP4 1534 kb)

Supplementary file6 (MP4 251 kb)
