# Supplementary material for: Smart Cellulose-Based Janus Fabrics with Switchable Liquid Transportation for Personal Moisture and Thermal Management
Source: Nanomicro Lett. 2024 Sep 26;17:14. doi: 10.1007/s40820-024-01510-5 (PMC11427652; doi:10.1007/s40820-024-01510-5)
Supplement: Supplementary file 1 — Supplementary file1 (DOCX 4329 KB) [file 40820_2024_1510_MOESM1_ESM.docx]

Supporting Information for

**Smart Cellulose-Based Janus Fabrics with Switchable Liquid Transportation for Personal Moisture and Thermal Management**

Jianfeng Xi^1^, Yanling Lou^1^, Liucheng Meng^1^, Chao Deng^4^, Youlu Chu^1^, Zhaoyang Xu^2^, Huining Xiao^3^, Weibing Wu^1,^ *

^1^Jiangsu Co-Innovation Center of Efficient Processing and Utilization of Forest Resources, International Innovation Center for Forest Chemicals and Materials, Jiangsu Provincial Key Lab of Sustainable Pulp and Paper Technology and Biomass Materials, Nanjing Forestry University, Nanjing 210037, P. R. China

^2^College of Materials Science and Engineering, Nanjing Forestry University, Nanjing 210037, P. R. China

^3^Department of Chemical Engineering, University of New Brunswick, Fredericton, NB E3B 5A3, Canada

^4^Macromolecular Chemistry and Bavarian Polymer Institute, University of Bayreuth, 95440 Bayreuth, Germany

*Corresponding author. E-mail: [wbwu@njfu.edu.cn](mailto:wbwu@njfu.edu.cn) (Weibing Wu)

**Supplementary Figures and Tables**

**Table S1** Hydrophilic/hydrophobic composition of different fabrics

| Sample name | Hydrophobic side | Hydrophilic side |
| --- | --- | --- |
| NCFB | √ | ╳ |
| NCFH | ╳ | √ |
| NCFBM | EC ethanol solution with a total mass 4 times the mass of CF | √ |
| JCF | √ | √ |

Note: √ means that this part of the experiment has been processed, and ╳ means that this part of the experiment has not been processed.

g-C_3_N_4_ nanosheets were prepared by urea thermal polymerization. Briefly, the urea was heated to 550 °C at a rate of 2 °C /min and kept for 4 h. After cooling, light-yellow g-C_3_N_4_ nanosheets were obtained.

The g-C_3_N_4_ nanosheets were successfully obtained by urea thermal polymerization. As shown in Fig. S1a, the obtained g-C_3_N_4_ nanosheets present a dense multi-layer stacked structure. In addition, CNF is between 4-10 nm in diameter and 1-3 μm in length (Fig. S1b).


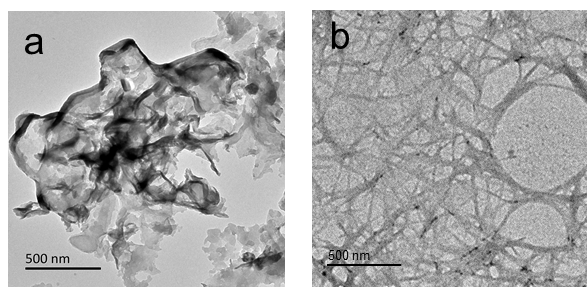


**Fig. S1** TEM images of **a** g-C_3_N_4_ nanosheets and **b** CNF

As can be seen from the floor plan, after the fabric is sprayed by EC, EC acts like glue to bind the fibers on the yarn together, and it is obvious that there is a layer of coating on the yarn (Fig. S2). The SEM image of the cross section also shows that the fibers on the hydrophobic side are glued to each other. Because the CNF just spray on the fiber surface, so from the cross-sectional SEM, the NIPAM modified layer and hydrophilic layer without obvious changes. However, it can be clearly seen from the floor plan that the surface roughness of the hydrophilic layer is improved.

**
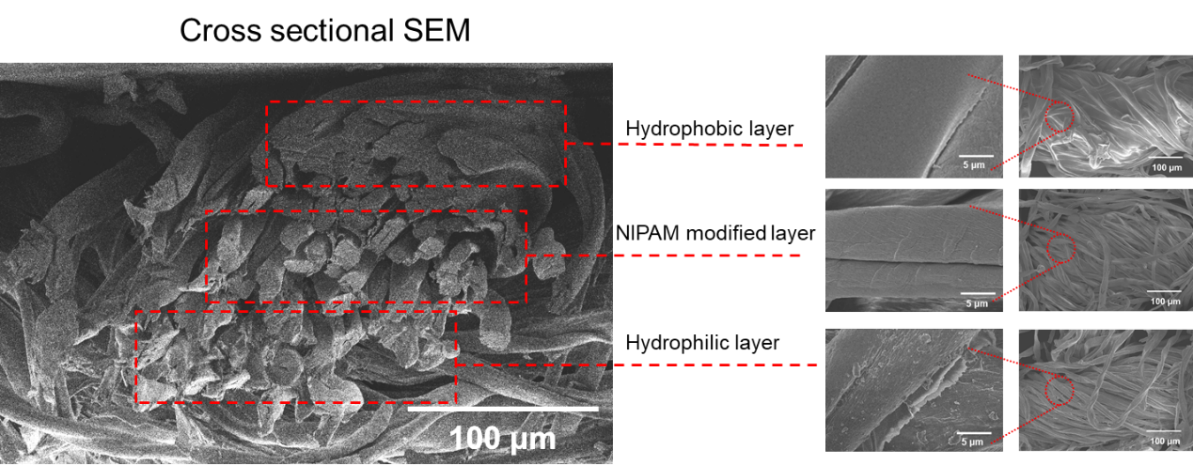
**

**Fig. S2** SEM images of the yarn

Each sample has the main infrared characteristic peaks of CF (Fig. S3a), such as the peak around 3320 cm^-1^, which corresponds to the stretching vibration of -OH, and the peak at 2920 cm^-1^, which corresponds to the stretching vibration of -C-H. The NCF modified by NIPAM appears two new infrared characteristic absorption peaks, the stretching vibration of amide group appeared at 1546 cm^-1^, and the stretching vibration of C-N appeared at 1450 cm^-1^. In addition, the infrared absorption peak of carboxyl group appeared in JCF due to CNF spray and citric acid crosslinking modification. The surface elements of the fabrics were detected by X-ray photoelectron spectroscopy (XPS). The photoelectron peaks at 285, 399, and 531 eV correspond to C1s, N1s and O1s, respectively (Fig. S3b). In comparison with CF, the NCF shows new photoelectron peak of N element and the relative content of N element reached 3.95% (Table S1), indicating that NIPAM was successfully introduced into CF. At the same time, the presence of g-C_3_N_4_ nanosheets increases the relative content of N element in JCF to 5.90%.


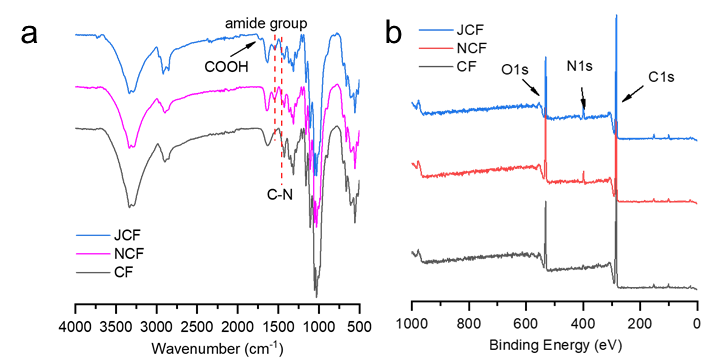


**Fig. S3 a** FT-IR spectra and **b** XPS of the fabrics

**Table S2** Elemental composition characterized by XPS

| **Sample name** | **O 1s/%** | **N 1s/%** | **C 1s/%** |
| --- | --- | --- | --- |
| CF | 20.74 | 0.00 | 79.26 |
| NCF | 18.15 | 3.95 | 77.90 |
| JCF | 17.11 | 5.90 | 76.99 |

With the extension of time, the WCA of the hydrophilic side of JCF reaches 0° when the water drops behind 4s, while it only takes 3 s after the addition of g-C_3_N_4_ (Fig. S4a-b). The presence of g-C_3_N_4_ further improves the hydrophilicity of the hydrophilic side.


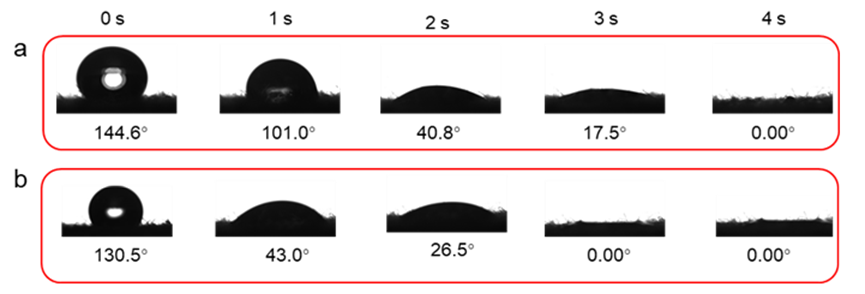


**Fig. S4** **a** WCA of JCF-without C_3_N_4_ and **b** JCF hydrophilic side of changes with time

NCF has different wettability at different temperatures (Fig. S5a). After multiple temperature cycles, the NCF exhibits reversible temperature responsiveness (Fig. S5b). For JCF sprayed with EC and CNF, they only cover the surface of the yarn and do not penetrate into the interior of the yarn. Therefore, spraying EC and CNF does not affect the temperature responsiveness of the yarn. We verified this by measuring the WCA decline rate on the hydrophobic side of JCF at different temperatures. As the temperature increases, the contact angle decreases at a faster rate (Fig. S5c).


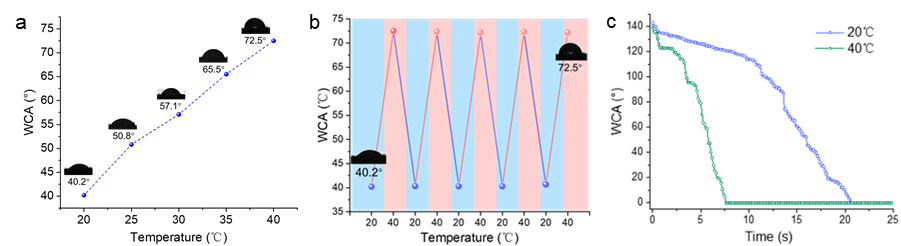


**Fig. S5 a** NCF temperature response wettability. **b** NCF reversible temperature response wettability. **c** The change of JCF hydrophobic side WCA with time at different temperatures


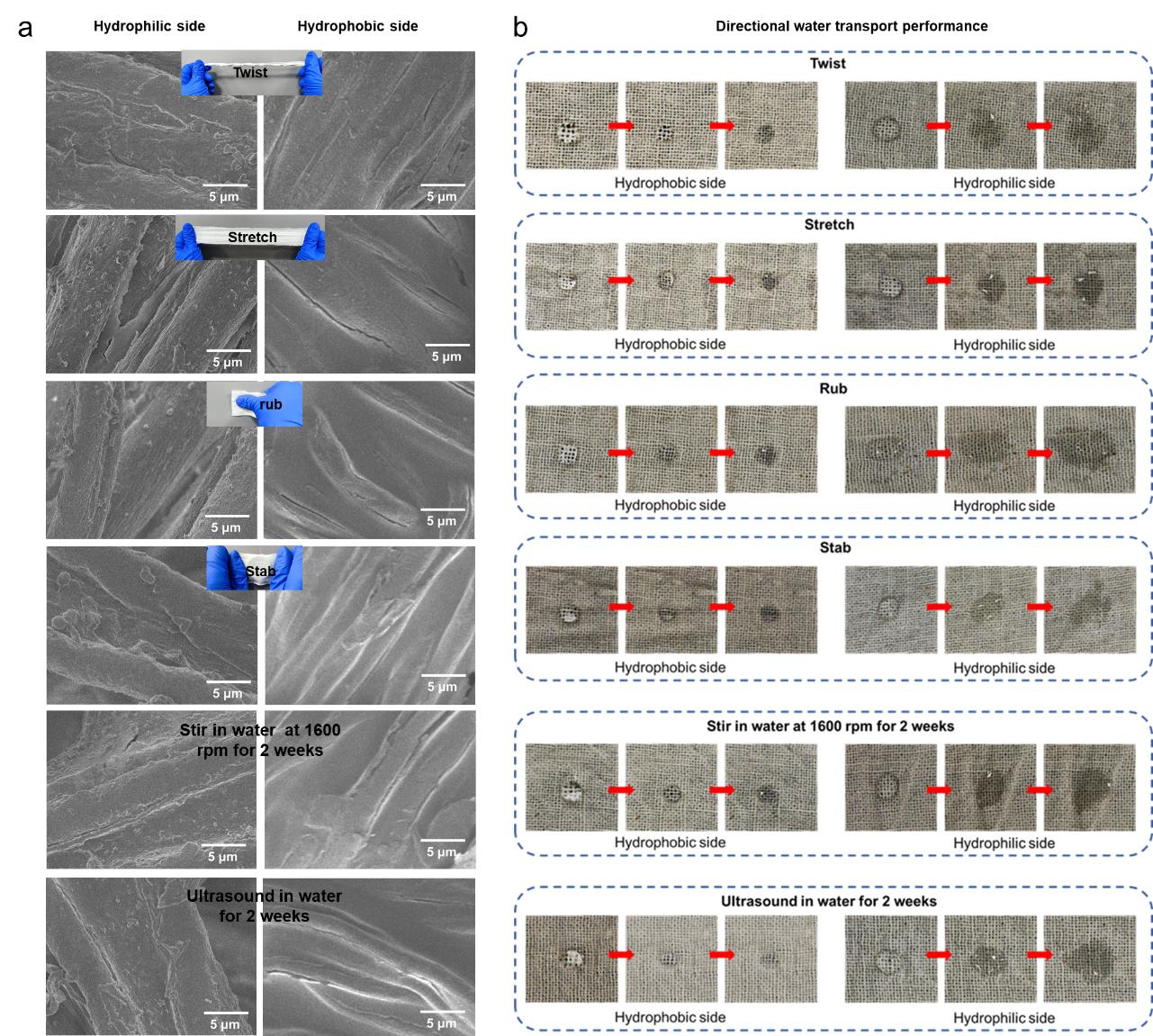


**Fig. S6** **a** Surface morphologies and **b** directional water transport performance of JCF under fabric deformation, after 1600 rpm for two weeks, and after 100 W ultrasonic treatment for two weeks


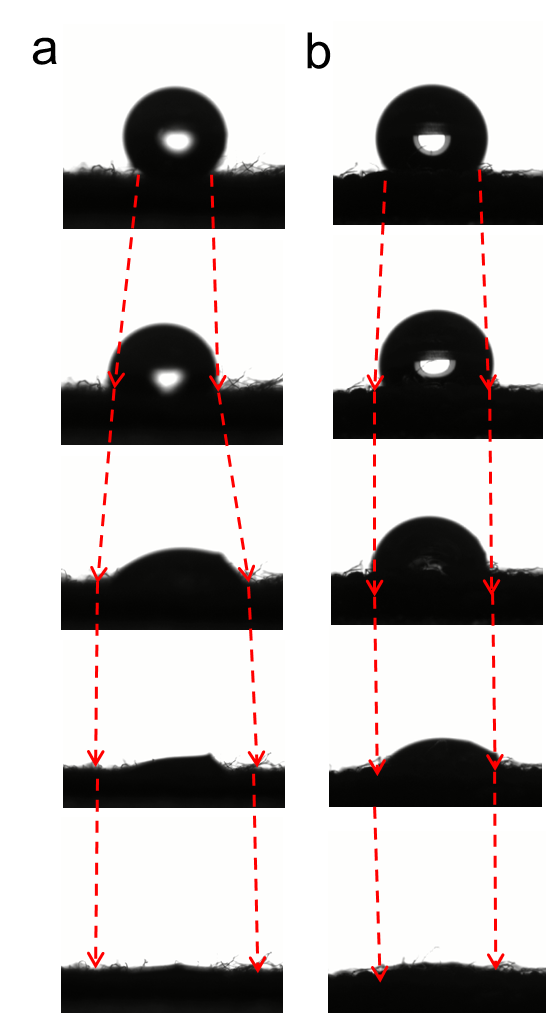


**Fig. S7** Changes in **a** water contact angle of droplets on the hydrophilic surface and **b** hydrophobic surface


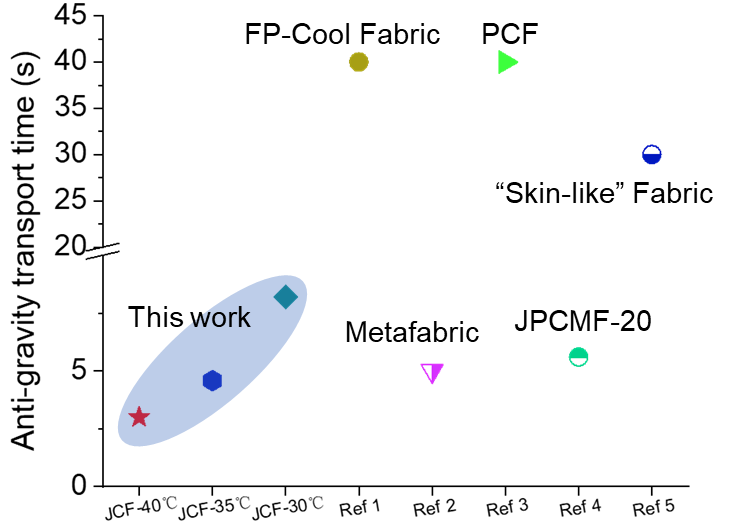


**Fig. S8** The antigravity water transport speed comparison of other Janus fabric

The air permeability of JCF shows a slight decline due to the decrease in air permeability after NIPAM modification. However, this reduction is only 9.82% compared to CF (Fig. S9).


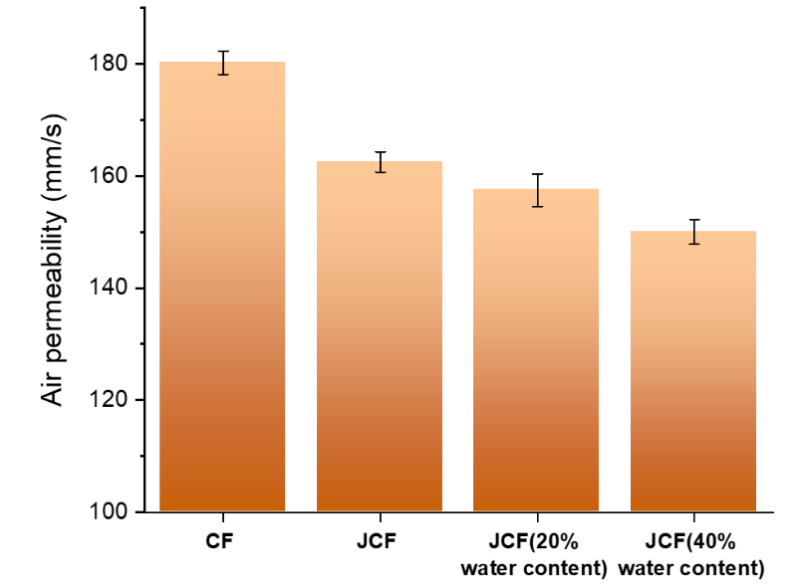


**Fig. S9** Air permeabilities of CF and JCF under different water content


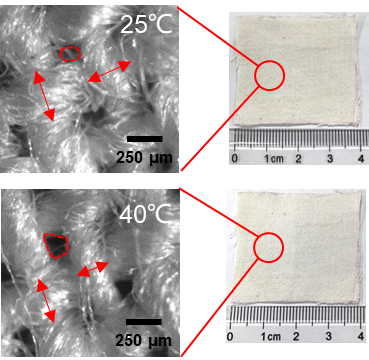


**Fig. S10** Pore size change of fabric at 25 and 40 °C


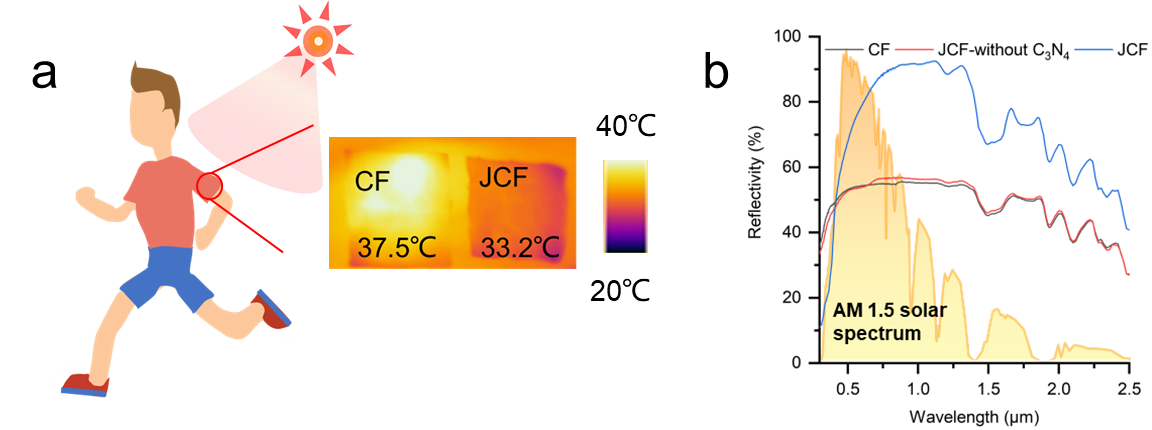


**Fig. S11** **a** Infrared images taken when fabrics were applied to skin at 40 ℃ under sunlight. **b** The reflectivity of fabrics


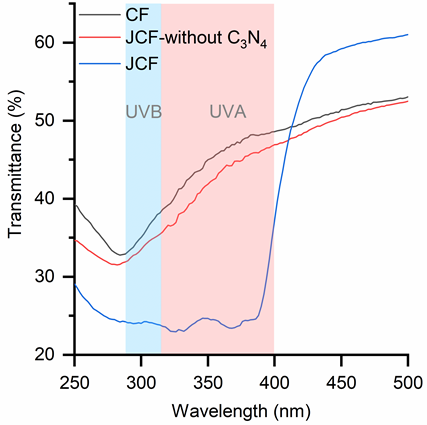


**Fig. S12** UV–vis transmission spectra of fabrics


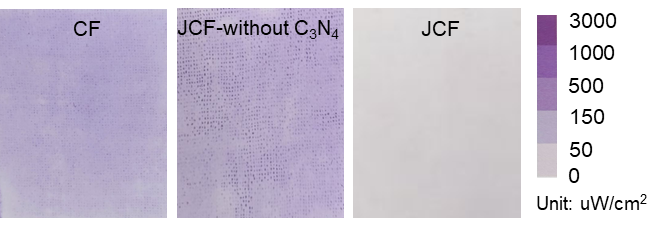


**Fig. S13** UV transmittance of fabric characterized by UV-induced color change card


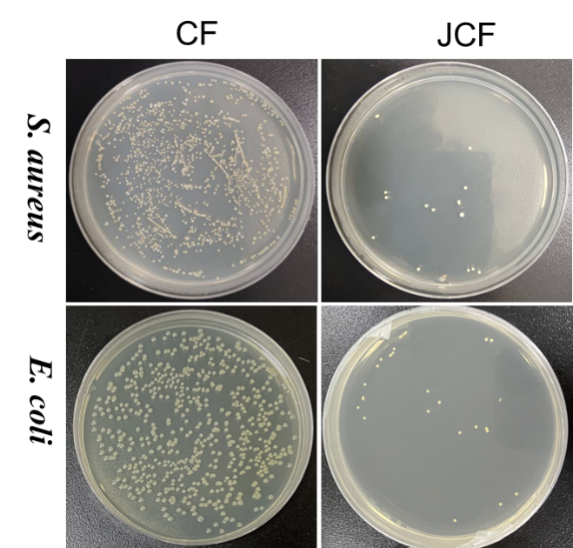


**Fig. S14** Photodynamic antimicrobial performance of JCF
